# Coacervate Thermoresponsive Polysaccharide Nanoparticles as Delivery System for Piroxicam

**DOI:** 10.3390/ijms21249664

**Published:** 2020-12-18

**Authors:** Dorota Lachowicz, Agnieszka Kaczyńska, Anna Bodzon-Kulakowska, Anna Karewicz, Roma Wirecka, Michał Szuwarzyński, Szczepan Zapotoczny

**Affiliations:** 1 Academic Centre for Materials and Nanotechnology, AGH University of Science and Technology, al. A. Mickiewicza 30, 30-059 Krakow, Poland; roma.wirecka@gmail.com (R.W.); szuwarzy@agh.edu.pl (M.S.); 2 Faculty of Chemistry, Jagiellonian University, Gronostajowa 2, 30-387 Krakow, Poland; agnieszka.kaczynska01@gmail.com (A.K.); karewicz@chemia.uj.edu.pl (A.K.); zapotocz@chemia.uj.edu.pl (S.Z.); 3 Department of Analytical Chemistry and Biochemistry, Faculty of Materials Science and Ceramics, AGH University of Science and Technology, al. A. Mickiewicza 30, 30-059 Krakow, Poland; anna.bodzon-kulakowska@agh.edu.pl; 4 Faculty of Physics and Applied Computer Science, AGH University of Science and Technology, al. A. Mickiewicza 30, 30-059 Krakow, Poland

**Keywords:** drug delivery system, self-organization, hydroxypropyl cellulose, curdlan, piroxicam

## Abstract

Low water solubility frequently compromises the therapeutic efficacy of drugs and other biologically active molecules. Here, we report on coacervate polysaccharide nanoparticles (CPNs) that can transport and release a model hydrophobic drug, piroxicam, to the cells in response to changes in temperature. The proposed, temperature-responsive drug delivery system is based on ionic derivatives of natural polysaccharides—curdlan and hydroxypropyl cellulose. Curdlan was modified with trimethylammonium groups, while the anionic derivative of hydroxypropyl cellulose was obtained by the introduction of styrenesulfonate groups. Thermally responsive nanoparticles of spherical shape and average hydrodynamic diameter in the range of 250–300 nm were spontaneously formed in water from the obtained ionic polysaccharides as a result of the coacervation process. Their morphology was visualized using SEM and AFM. The size and the surface charge of the obtained objects could be tailored by adjusting the polycation/polyanion ratio. Piroxicam (PIX) was effectively entrapped inside the nanoparticles. The release profile of the drug from the CPNs-PIX was found to be temperature-dependent in the range relevant for biomedical applications.

## 1. Introduction

Nanoparticulate systems display many advantages as carriers in drug delivery applications [1,2]. When applied parenterally, they increase the bioavailability of the compounds, which are either poorly soluble in aqueous media or characterized by low stability. This allows for the reduction of a necessary dose, increasing both the efficiency and safety of the therapy [3]. Incorporating a drug into the nanoparticulate system may also allow improving its biodistribution [4]. The nanocarrier may also allow for controlled release of the active compound or for its targeted delivery. Such systems are often applied in ophthalmic delivery, as they offer longer residence times at the site of the cul-de-sac. Nanoparticles are less irritant to the eye than larger particles and may improve the saturation and inherent solubility of hydrophobic drugs in lachrymal fluids [5].

Nanoparticles formed as a result of coacervation constitute a unique group of drug carriers [6,7,8]. Coacervates are usually defined as micro- or nanospheres formed by the assorted organic biomolecules that are associated with weak interactions [9,10,11]. Self-assembly of the oppositely charged polyions is one of the most convenient coacervation methods used to obtain nanoparticles [12,13], which allows obtaining nanoparticulate drug delivery systems without the use of potentially harmful cross-linking agents or surfactants. It also allows for encapsulating sensitive drugs in mild conditions [8]. Among such coacervate systems, the nanoparticles composed of physically cross-linked stimuli-responsive polymers have unique properties [14], such as the responsiveness to environmental stimuli and the ability to accommodate a wide range of active molecules. The purpose of the current study was to obtain and characterize the coacervate nanoparticulate system based on the formation of polyion complex between two polysaccharide derivatives: cationic curdlan (C-CUR) and anionic hydroxypropyl cellulose (A-HPC), and to evaluate its usefulness in the delivery of a poorly soluble drug, piroxicam.

Hydroxypropyl cellulose (HPC) is a thermoresponsive, non-toxic, biodegradable, and hydrophilic polysaccharide, which is characterized by phase behavior and ease of production [7,15]. In aqueous solutions, HPC exhibits so-called low critical solution temperature (LCST) at ca. 43 °C, however, this LCST value can be modified by the addition of salts or polyions [16]. We have modified HPC by introducing negatively charged styrenesulfonate groups. As a counter polyion, we have chosen cationically modified curdlan. Curdlan is a water-insoluble linear beta-1,3-glucan, built from the β-(1,3)-linked glucose residues connected by β-1,3-glucosidic linkages. It forms elastic gels upon heating in aqueous suspensions [17]. In order to increase its water solubility and to introduce the charge, we have modified curdlan’s chains with trimethylammonium groups, obtaining a cationic polyelectrolyte (C-CUR). The choice of CUR as a component of the self-organizing system was dictated by its ability to form gels in response to temperature change and by its exceptional biological activity. CUR is known to possess anti-inflammatory, anti-tumor, and immunomodulating properties [18]. CUR, like other β-glucans, is recognized by dectin-1 receptors on the surface of macrophages [19] and can convert M2 polarized tumor-associated macrophages (TAMs) to M1 phenotype [20], thus may prevent cancer progression and metastasis [21]. CUR was also found to have an immunomodulatory effect in the fungal keratitis, and thus the nanocarriers based on CUR may be well suited for the treatment of this severe corneal disease, which can lead to sight disability [22].

We used piroxicam as the drug of choice. It belongs to the family of nonsteroidal anti-inflammatory drugs, oxicams, and is an unselective inhibitor of cyclooxygenases COX-1 and COX-2. Piroxicam is widely used to treat inflammation, including ophthalmic formulations, e.g., in controlling inflammation after cataract surgery [23], however, due to its poor solubility, it requires a suitable carrier. Recent studies have also shown the potential of piroxicam in the treatment of nonmelanoma skin cancer [24] and bladder cancer [25] by downregulating COX-1, COX-2, and prostaglandins production, but also through the induction of cell apoptosis and suppression of metalloproteinase-2 activity [24].

We believe that the proposed coacervate thermoresponsive nanoparticulate system based on A-HPC and C-CUR will be suitable for the delivery of anti-inflammatory and anti-tumor drugs. It may be easily obtained through simple mixing of the components in water, with no necessity for potentially harmful solvents, surfactants, or cross-linkers. The thermo-responsiveness of the system may be advantageous in selected applications (e.g., in the drug delivery to locally elevated temperature sites, e.g., inflamed or undergoing hyperthermia treatment). The nanometric size of the particles will bring significant benefits in either parenteral or ophthalmic delivery. The unique biological activity of CUR may prove important in cancer therapy, or when its immunomodulatory properties can be exploited. As a proof-of-concept, we have also entrapped piroxicam in the proposed A-HPC/C-CUR nanoparticles. The obtained system, due to the combined properties of CUR and the drug, may have potential in anti-cancer therapy.

## 2. Results and Discusion

### 2.1. Cationic Curdlan—Synthesis and Characterization

**Figure 1:**
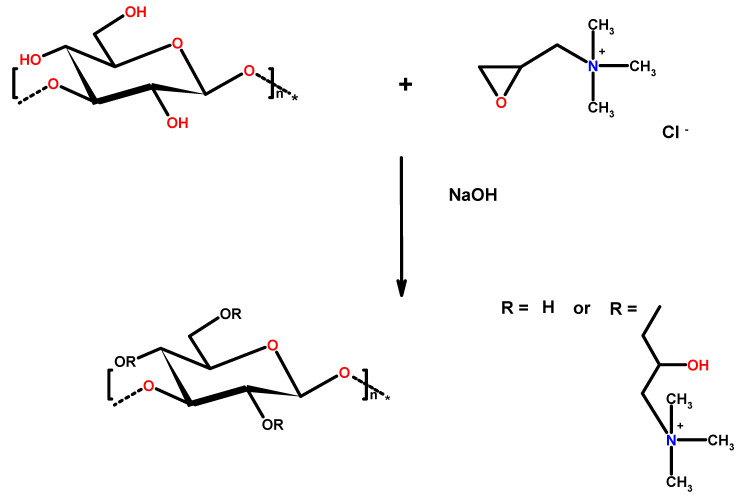
Scheme of cationic curdlan (C-CUR) synthesis.

The cationic derivative of curdlan (C-CUR) was synthesized by reacting curdlan with glycidyltrimethylammonium chloride (GTMAC) (Figure 1). The hydroxyl groups in the structure of curdlan can react with GTMAC under alkaline conditions.

**Figure 2:**
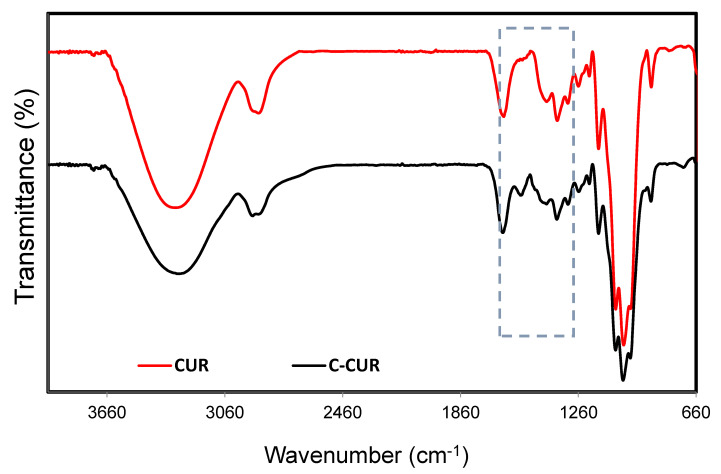
FT-IR spectra of CUR (red) and C-CUR (black).

Figure 2 shows the ATR-FTIR spectra obtained for curdlan (CUR) and its derivative (C-CUR). A broad band in the range of 3050–3500 cm^−1^, which can be assigned to the stretching vibrations of the polysaccharide’s hydroxyl groups, was observed in both spectra. Moreover, bands at 2884, 1366, and 1068 cm^−1^ can be assigned to the stretching vibration of −CH_2_ groups. The important changes in the CUR spectrum after modification were observed in the region between 1300–1500 cm^−1^, which was marked with a frame (see Figure 2). The most significant differences between curdlan and its cationic derivative can be noticed between 1461 and 1500 cm^−1^, corresponding to the ethyl and methyl groups bound to the quaternary nitrogen in the trimethylammonium group [26,27]. ^1^H NMR analysis was also used to confirm the structure of C-CUR. The additional signal at 3.02 ppm was observed in the C-CUR spectrum (Figure S1), originating from the nine protons of the [N(CH_3_)_3_]^+^ group. Moreover, signals at 1.2–1.3 ppm and at 1.8–2.0 ppm were observed, which can be attributed to the −CH_2_ and −CH protons, through which trimethylammonium groups were attached to curdlan. Based on the intensity ratio of the signals of the six ring-skeleton protons in the range of 3.00–3.75 ppm and the signal of nine [N(CH_3_)_3_]^+^ protons at 3.02 ppm, the degree of substitution (DS) was calculated to be 8.9 ± 2.0%. The DS for C-CUR was also confirmed using a method described by Cho [28], based on the conductivity measurements performed in the presence of varying amounts of AgNO_3_. The degree of CUR modification was estimated to be 11.2 ± 1.1% (Table S1).

**Figure 3:**
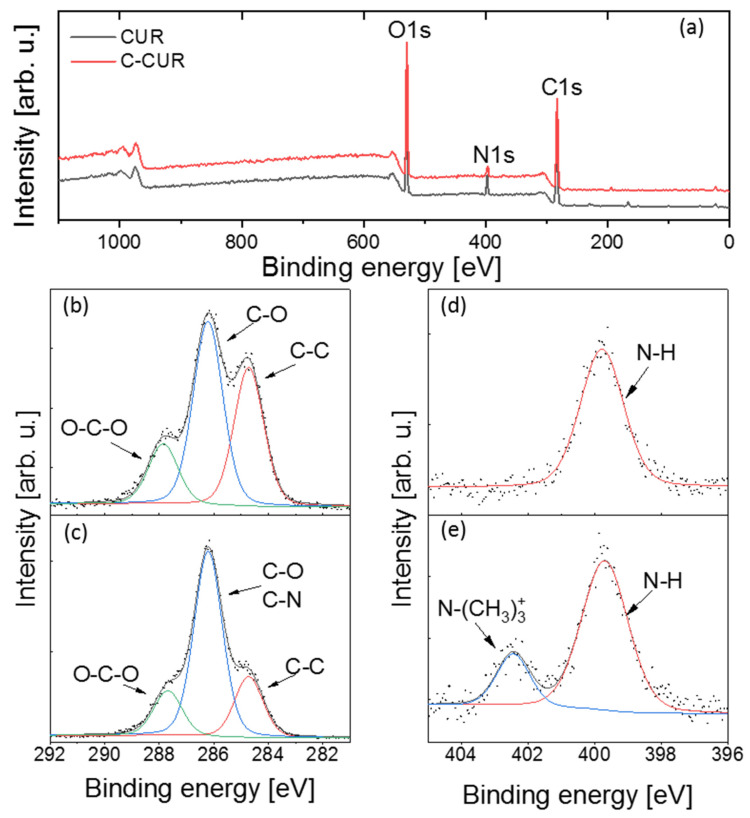
XPS spectra of (**a**) CUR and C-CUR survey, C1s spectrum of CUR (**b**), and C-CUR (**c**), N1s spectrum of CUR (**d**), and C-CUR (**e**).

Next, XPS analyses were performed for CUR and C-CUR in order to further confirm the success of the grafting process (see Figure 3). The C1s core-level spectra for both polymers consist of three lines at 284.8 ± 0.1, 286.3 ± 0.1, and 287.9 ± 0.1 eV, corresponding to C–C, C–O (and C–N), and O–C–O bonds, respectively [29]. In the C-CUR spectrum, the ratio of the C–O band area to the integrated area of other carbon bands is larger than in the spectrum of CUR, implying the successful modification of the polymer. In the O1s spectra, no differences were observed between the pristine polymer and its cationic modification, indicating no changes in the main polymer chain that might occur as the result of the modification procedure. The presence of N–H line (399.8 ± 01 eV) in the N1s region can be explained by a natural origin of the polymer, and a possible presence of impurities in the sample [30], while the occurrence of an additional line in C-CUR N1s spectrum with the binding energy of 402.5 eV ([N(CH_3_)_3_]^+^) [31] confirms successful cationic modification.

### 2.2. Preparation of Thermoresponsive Coacervate Polysaccharide Nanoparticles (CPNs)

Thermosensitive nanoparticles were formed in an aqueous solution as a result of the electrostatic polycation-polyanion interactions. As a counter-polyion to the synthesized cationic C-CUR, we used the anionic derivative of hydroxypropyl cellulose (A-HPC), which was obtained following our previous report [32]. The degree of substitution (DS) was determined to be equal to 29 ± 9% (Table S2).

To optimize the formation procedure and obtain stable colloids, a series of systems was prepared by varying the ratio of the two polyelectrolytes used. The nanoparticles were formed spontaneously in the coacervation process, directly after mixing the solutions of both polyelectrolytes. The formed nanoparticles’ hydrodynamic diameters were investigated using dynamic light scattering (DLS), while their zeta potentials were measured with the same apparatus using Electrophoretic Light Scattering (ELS) methodology. The results are presented in Table 1. All tested systems were stable enough to obtain reproducible results.

The comparison of the SEM images of various obtained particles revealed that the size and morphology of the synthesized structures depended strongly on the weight ratio of the polyelectrolytes used (Figure S2). Large, irregular microparticles were obtained when a 1:1 ratio was used. When the C-CUR/A-HPC ratio was 1:10, the resulting particles had a size of 300–350 nm and appeared to be clusters of several smaller particles of 30–50 nm. The 1:25 C-CUR/A-HPC ratio resulted in the formation of regular, spherical nanoparticles, which had diameters ranging from 150 to 250 nm, and were characterized by the lowest dispersity of all the systems studied. This system was therefore selected for further investigations.

**Figure 4:**
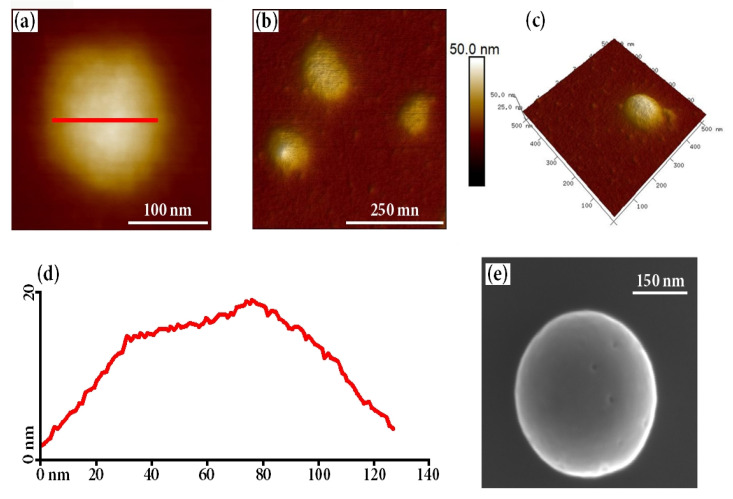
(**a**–**c**) AFM images of C-CUR/A-HPC nanoparticles structures (at C–CUR: A-HPC ratio of 1:25); (**d**) C-CUR/A-HPC cross-section and (**e**) SEM image of obtained nanoparticle.

The selected nanoparticulate system was then characterized using atomic force microscopy (AFM). The obtained images, shown in Figure 4, confirmed that indeed spherical particles were obtained, which had a moderately rough surface and an average diameter of ca. 150 nm. The size (130–175 nm) of the obtained particles was slightly lower than that obtained from DLS measurements, which is expected considering the lack of a surrounding solvating shell. The obtained cross-section profiles clearly indicated low roughness of the particles’ surface.

In the next stage of the work, the thermosensitivity of the synthesized nanoparticles was tested. The upper critical solution temperature (UCST) value was first determined for the modified (C-CUR) and unmodified (CUR) curdlan, and the lower critical solution temperature (LCST) value was determined for the modified (A-HPC) and unmodified (HPC) hydroxypropyl cellulose. Finally, LCST value was also found for the C-CUR/A-HPC (1:25) nanoparticulate system. The results are presented in Table 2.

No difference was found between the LCST values determined for modified and unmodified HPC—both values were estimated to be around 43 °C.

**Figure 5:**
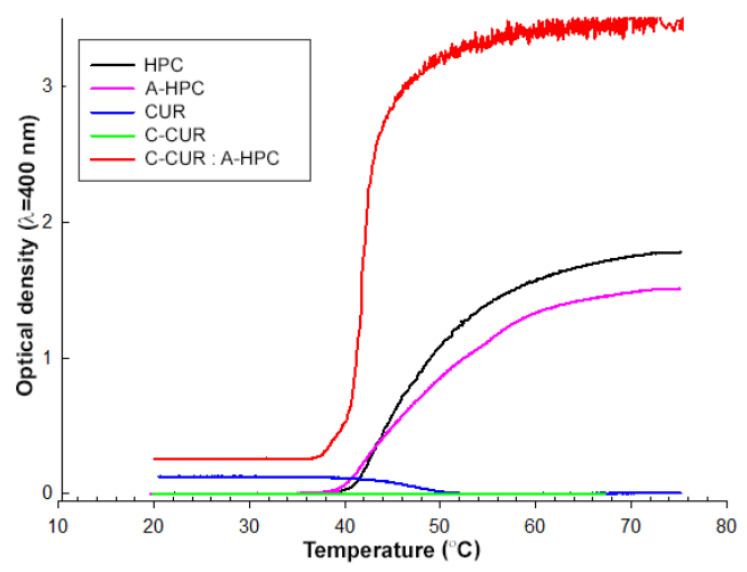
Temperature dependence of the optical density of CUR, HPC, their derivatives, and obtained nanoparticulate systems in water.

UCST was observed for CUR, and its value was determined to be 47 °C. Unlike the unmodified polymer, the cationic derivative of curdlan, C-CUR, showed no UCST at the same concentration (0.125 mg/mL), which is most likely related to an increase in its hydrophilicity due to the chain modification. Based on the results obtained for both ionic derivatives, one could expect that C-CUR/A-HPC nanoparticles may show LCST, similarly to A-HPC. This was confirmed experimentally, as shown in Figure 5. The LCST of the aqueous suspension of the nanoparticles was observed at 41 °C. Based on this observation, further studies on the thermo-responsiveness of the obtained C-CUR/A-HPC (1:25) nanoparticles in aqueous media were performed.

The results showing the dependence of the hydrodynamic diameter of the obtained nanoparticles on the temperature are shown in Table 3. A decrease in the size and polydispersity index of the nanoparticles was registered in the temperature range from 21 °C to 39 °C, and then at 42 °C, an increase in the hydrodynamic diameter was clearly observed. At the 45 °C, the size of the particles started to grow fast, suggesting that the aggregation process took place.

### 2.3. Binding Constant of Piroxicam to A-HPC

**Figure 6:**
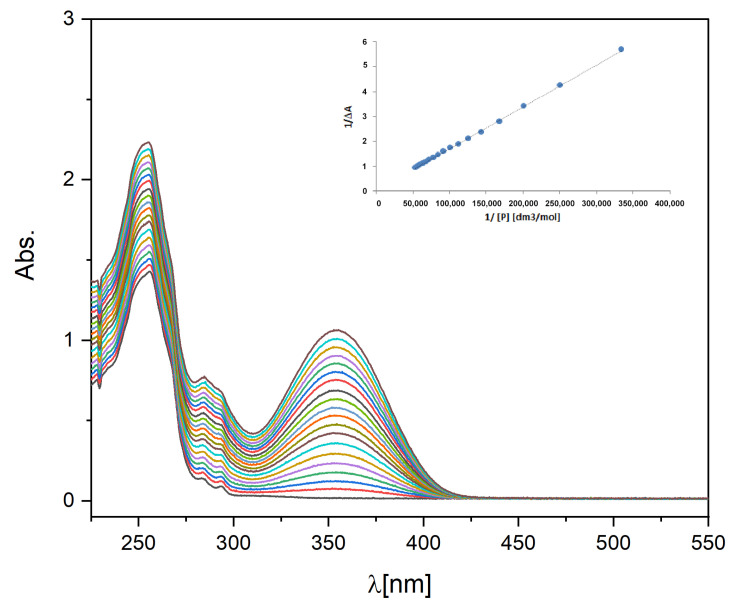
UV-Vis spectra of the A-HPC water solutions in the presence of different concentrations of piroxicam (concentration of piroxicam in the range of 0 to 0.09 (mM); c_A-HPC_ = 1 (mg/mL)). The insert shows 1/∆A plot as a function of 1/[P] and the linear fit.

Piroxicam’s affinity to A-HPC was evaluated by determining the binding constant, K_a_. The Benesi–Hildebrand method allows the investigation of the one-to-one binding process between host and guest molecules using spectrophotometric measurements [33,34,35]. K_a_ value was determined in water. Figure 6 shows a set of UV-Vis absorption spectra of the A-HPC solution (c = 1 mg/mL) with various amounts of piroxicam added. The insert shows a double inverse plot (1/∆A versus 1/[P]) and data fitting according to the Benesi–Hildebrand model. Based on the obtained fit, the K_a_ value was determined to be 2.6 × 10^3^ (M^−1^). This value is one order of magnitude lower than the K_a_ value determined in the literature for the BSA/piroxicam delivery system [36]. However, this value should still be sufficient to transport piroxicam via a carrier having A-HPC in its structure.

### 2.4. Piroxicam-Loaded C-CUR/A-HPC (1:25) Nanoparticles (CPNs-PIX)

Piroxicam was trapped inside the nanoparticles as a result of the self-assembly process of C-CUR and A-HPC. First, a methanolic solution of piroxicam was added dropwise to the aqueous solution of A-HPC. Then methanol was evaporated, and the C-CUR solution was added while stirring.

The entrapment efficiency (EE) and loading efficiency (LE) for the NPs-PIX system were determined to be: EE = 94.80%, and LE = 0.73%.

### 2.5. Piroxicam Release

**Figure 7:**
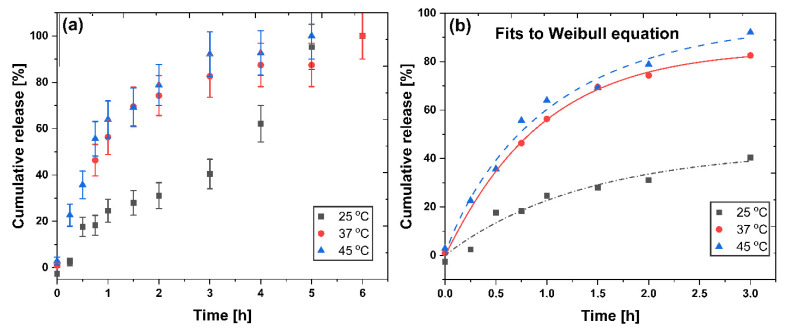
Piroxicam release profiles at 25, 37, and 45 °C (pH = 7, water) from CPNs-PIX system (**a**). Fitting the piroxicam release data to the Weibull release model (**b**).

The release profiles of the drug from CPNs-PIX system in water (pH 7.0) at 25, 37, and 45 °C are presented in Figure 7. No burst release effect was observed in all temperatures studied. The observed release is temperature-dependent, and the higher the temperature, the faster the release. This can be easily explained by the well-known influence of temperature on the diffusion coefficient, suggesting that piroxicam release is diffusion driven. To further analyze the release mechanism, the experimentally determined profile data obtained at various temperatures were fitted to the well-known theoretical models. The Higuchi [37,38], Peppas [39,40], and Weibull [41,42] models are widely used to characterize the release process and are described by the following Equations (1)–(3):

(1) $$Higuchi\;Q=k_{H}\cdot t^{\frac{1}{2}}$$

where ***Q***—the amount of piroxicam released; ***k_H_***—Higuchi constant; ***t***—time.

(2) $$Peppas\;Q=a\cdot t^{n}$$

where ***Q***—the amount of piroxicam released; *a*—kinetic constant; *n*—exponent characterizing the diffusion mechanism; ***t***—time.

(3) $$Weibull\;Q=a\cdot (1-exp\left(-\left(kt{)}^{b}\right)\right)$$

where ***Q***—the amount of piroxicam released; *a*—kinetic constant; *b*—exponent characterizing the diffusion mechanism; ***t***—time.

The results of the fitting of release data to the Higuchi, Peppas, and Weibull models are presented in Table 4. Based on relatively good fitting to the Higuchi model, it may be concluded that in all three temperatures studied, a short-time approximation confirms a diffusion-driven release mechanism. Further analysis of the Peppas model suggests an anomalous (non-Fickian) diffusion from the spherical matrix for the drug release observed at room temperature (0.43 < n < 1), while at 37 °C a Fickian diffusion is predicted. Interestingly, the n value obtained from the Peppas fit at 45 °C is 0.43, a limit above which the release mechanism becomes non-Fickian. It shows the influence of the LCST value of the nanoparticles on the release process—above LCST, the release may become non-Fickian.

Based on the results in Table 4, the best fit was obtained using the Weibull model, which is a long-term approximation. Papadopoulou et al. [41] have shown that there is a correlation between the value of b in the Weibull equation and the type of diffusional mechanism observed. The b values obtained in the case of all the temperatures studied were in the range of 0.90–0.99, for which the Weibull model predicts a combined mechanism of Fickian diffusion and Case II transport. Thus, we may conclude that the release mechanism initially depends on temperature and LCST of nanoparticles, but in the long-term approximation, it can be described by a similar, combined mechanism in all temperatures studied.

### 2.6. Biological Studies

**Figure 8:**
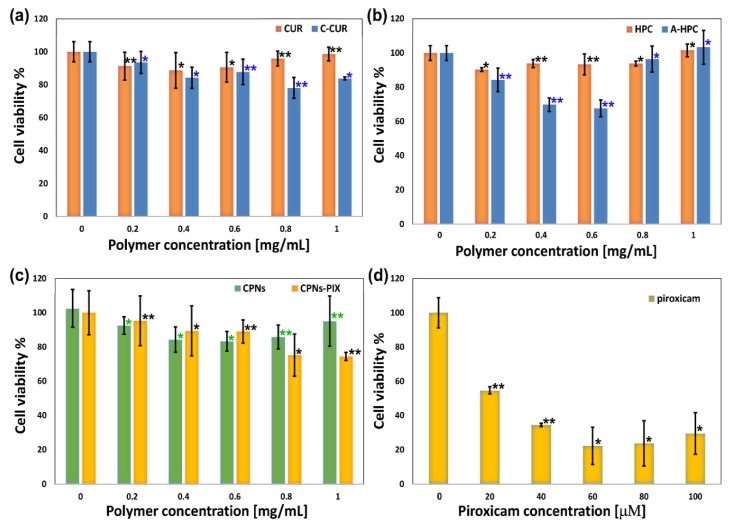
MTT assay test performed for NIH3T3 cells incubated for 24 h with (**a**) CUR and C-CUR; (**b**) HPC and A-HPC; (**c**) polysaccharide-based nanoparticulate systems (CPNs and CPNs-PIX), and (**d**) free piroxicam. Cell viability is presented as the percent of the control (cells incubated without any polymer system added). Error bars represent the mean and standard deviation of the individual experiments performed in triplicate (*n* = 3). * *p* < 0.05, ** *p* < 0.01 compared with the control (0 µM or 0 mg/mL) group.

In the final stage of the study, the cytotoxicity of the obtained CPNs and CPNs-PIX systems was examined, as well as the cytotoxicity of both polysaccharide components of the system (C-CUR, A-HPC), and their unmodified counterparts (CUR, HPC). The viability of mouse fibroblasts (NIH3T3 cell line) was tested using the MTT assay. Results are shown in Figure 8. Both C-CUR and A-HPC have a slight cytotoxic effect in concentrations above 0.4 mg/mL, while CUR, HPC, C-CUR/A-HPC nanoparticles are not toxic even at concentrations as high as 1 mg/mL. The cytotoxic effect caused by C-CUR is probably related to the presence of cationic groups in the polymer structure. An increase in the toxicity of cationic polymers was observed before [43,44] and may be related to the electrostatic interactions between the polycations and a negatively charged cell membrane. The toxic effect of C-CUR and A-HPC in nanoparticles is practically abolished as C-CUR binds electrostatically to A-HPC. The presence of piroxicam in CPNs-PIX slightly increased their toxicity (ca. 20% decrease in viability). However, this effect is much less pronounced than that observed for the unbound piroxicam.

**Figure 9:**
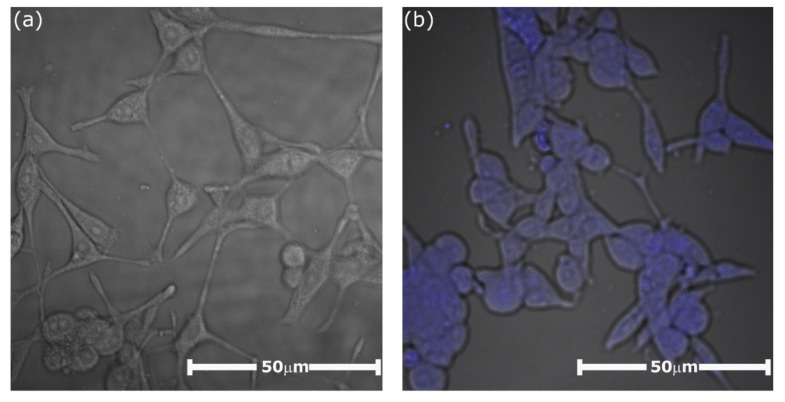
Confocal images obtained for murine fibroblast NIH3T3 cell line (60× objective, 2× zoom) recorded at 24 h after stimulation with (**a**) “empty” CPNs nanoparticles (control) and (**b**) CPNs-PIX system with encapsulated piroxicam. Excitation: 408 nm laser line; emission filter: 458 nm (barrier filter).

The penetration of the cells by the piroxicam entrapped in CPNs was examined for the NIH3T3 cell line using confocal microscopy. The observation was based on the fact that piroxicam exhibits a characteristic fluorescence in the visible range (emission maximum at 460 nm; excitation with the 408 nm laser line). The cellular uptake and the localization of piroxicam in the cells were studied 24 h after stimulation. In the captured images, presented in Figure 9, one can observe the intense blue emission from piroxicam in the cells that were stimulated with CPNs-PIX (Figure 9b). The emission was observed only inside the cells, with no visible blue signal in the extracellular environment. In contrast, blue emission was not present in the images of cells stimulated with CPNs system without piroxicam, as shown in Figure 9a. This confirms the successful and complete uptake of the drug entrapped in CPNs-PIX system by NIH3T3 cells.

## 3. Materials and Methods

### 3.1. Materials

Curdlan, CUR (from *Alcaligenes faecalis*), Hydroxypropyl cellulose, HPC (average Mw ~80,000, average Mn ~10,000), Glycidyltrimethylammonium chloride, GTMAC (≥90%), sodium p-styrenesulfonate, SSS (90%) were purchased from Sigma-Aldrich (Poznan, Poland). Spectroscopic grade methanol and oleic acid (p.a.) were purchased from POCH (Gliwice, Poland).

### 3.2. Synthesis of Ionic Polysaccharide Derivatives (C-CUR and A-HPC)

#### 3.2.1. C-CUR

1.0 g of CUR previously dissolved in 100 mL of deionized water was placed in a two-necked flask. The solution was intensively stirred on a magnetic stirrer at 60 °C for 30 min. Then 400 mg of NaOH dissolved in 1 mL of deionized water was added to the reaction mixture. After 15 min 7.0 mL of GTMAC (90%) was also introduced into the flask. The mixture was then vigorously stirred for 4 h at 60 °C. The reaction mixture was then cooled, moved to the dialysis tube (MWCO 12,000 Da cut-off, cellulose membrane), and dialyzed for 14 days against distilled water. The resulting polymer was isolated by lyophilization 0.952 g of product was obtained.

#### 3.2.2. A-HPC

The anionic derivative of hydroxypropyl cellulose (A-HPC) was obtained as a result of the grafting reaction of sodium p-styrene sulfonate (SSS) on the main polymer chain. The procedure for the preparation of this polymer, as described in our previous work [32].

### 3.3. Characterization of Modified Polysaccharides

XPS spectra were recorded using PHI 5000 Versa Probe II (ULVAC-PHI, Chigasaki, Japan) spectrometer with a monochromatic Al Kα radiation source (E = 1486.6 eV). To avoid a possible loading of the samples during measurements, dual-beam charge compensation was applied. High-resolution spectra were measured with an analyzer’s transition energy of 46.95 eV. All binding energies were corrected to the C–C line (at 284.8 eV). All measured spectra were deconvoluted using the PHI MultiPak software v9.3.03. The background subtraction was performed by the Shirley method. FT-IR measurements were carried out using Bruker IFS 48 (Bruker, Ettlingen, Germany) spectrometer equipped with a Diamond ATR accessory iD5 (Thermo Fisher Scientific, Waltham, MA, USA). Elementary analyses (EA) of the obtained polymers were performed with an Elemental Analyzer, Euro EA-3000 (EuroVector, Milano, Italy). 1 H NMR spectra were recorded using Bruker AMX 500 (Bruker Co., Rheinstetten, Germany) spectrometer (1 H, 500.14 MHz) at room temperature. The samples were dissolved in D_2_O.

### 3.4. Preparation of C-CUR/A-HPC ThermoResponsive Nanoparticles (CPNs)

Aqueous solutions of C-CUR (1 mg/mL) and A-HPC (10 mg/mL) were first prepared. Then, the C-CUR solution was added dropwise to the A-HPC solution with vigorous stirring until the desired volume ratio was obtained. Nanoparticles were formed immediately, as observed by an increase in the turbidity of the mixture. The solution was then mixed for 30 min, and next, the nanoparticles were centrifuged at 8800 rpm for 20 min at 4 °C, washed twice with distilled water, and re-suspended in 2 mL of distilled water.

### 3.5. Preparation of the Piroxicam-Loaded C-CUR/A-HPC System (CPNs-PIX)

200 µl of the methanolic solution of piroxicam (1 mg/mL) were added to 2.5 mL of A-HPC aqueous solution (10 mg/mL). The resulting solution was stirred at 35 °C for 30 min, while constantly purged with neutral gas (argon). 1 mL of aqueous C-CUR solution was then added to the A-HPC solution containing piroxicam while the mixture was vigorously stirred. NPs-PIX nanoparticles were formed immediately. The resulting mixture was then stirred for 30 min, and next the particles were separated by centrifugation (8800 rpm, 20 min, 4 °C). The purification was done the same way as for the CNPs system without piroxicam.

### 3.6. Morphology and Size of the Obtained Nanoparticles

The morphology of the obtained objects was examined using scanning electron microscopy (SEM) and atomic force microscopy (AFM). Aqueous suspensions of the obtained nanoparticles were placed on silicon substrates using a spin-coater and then dried. The samples were then coated with graphite using a Q150T E Plus ion sputtering machine (Quorum Technologies, Laughton, UK). The samples were applied to previously prepared silica plates coated with poly (allylamine hydrochloride) (PAH) using a spin-coater.

SEM observations were carried out using a Versa 3D FEG/SEM (FEI Company, Hillsboro, OR, USA) microscope operating at an accelerating voltage of 10 kV equipped with an SE (secondary electrons) detector. Atomic force microscope (AFM) images were captured using Dimension Icon XR atomic force microscope (Bruker, Santa Barbara, CA, USA) operating in the air in the PeakForce Tapping (PFT) mode, using standard silicon supports cantilevers with a nominal spring constant of 0.4 N/m, and tip radius below 8 nm.

Mean hydrodynamic diameters (d_Z_), polydispersity index (PDI) and ζ potentials were measured using a Malvern Nano ZS light scattering apparatus (Malvern Instrument Ltd., Worcestershire, UK) at 25 °C. The obtained values were a mean of five tests, and for each test, a mean from 3 different measurements (each with a mean value of 10 replicates) was determined. The data were analyzed using software provided by Malvern.

### 3.7. Determination of the Piroxicam-A-HPC-Binding Constant

The binding constant (*K*_a_) of piroxicam to the anionic A-HPC was determined based on the UV-Vis measurements using the spectroscopic titration technique. A detailed description of this technique was provided in our earlier work [45]. The changes in absorbance of the aqueous solution containing piroxicam in the concentration [*P*] ranging from 1 × 10^−6^ [M] to 8 × 10^−5^ [M] and the A-HPC concentration [*H*]_0_ = 1 mg/mL were investigated. Based on the Benesi–Hildebrand Equation (4), the binding constant (*K*_a_) was calculated.

(4) $$\frac{1}{\Delta A}=\frac{1}{b\varepsilon^{PH}{\left[H\right]}_{0}K_{a}}\cdot \frac{1}{\left(\left[P\right]\right)}+\frac{1}{b\varepsilon^{PH}{\left[H\right]}_{0}}$$

where Δ*A*—the difference between *A*— the absorbance at any point of the binding process and *A*_0_—the initial absorbance (without piroxicam present), *ε*—the differential extinction coefficient, and l—the optical path length.

Absorption measurements were made in a 1 cm quartz cuvette while maintaining the temperature at 25 °C and with continuous stirring. The spectra were recorded on a Thermo Scientific Evolution 220 spectrophotometer (Thermo Fisher Scientific, Waltham, MA, USA) equipped with a Peltier temperature controller (Thermo Fisher Scientific, Waltham, MA, USA).

### 3.8. Determination of Piroxicam Entrapment Efficiency (EE) and Loading Efficiency (LE)

The entrapment efficiency (EE%) of piroxicam in the CPNs-PIX system was determined using a sample palletization method [46] applying the following parameters: 15,000 rpm, 15 min, and 4 °C. The resulting precipitate was dispersed in water and then lyophilized. 3 mL of methanol were added to the 20 mg of the obtained sample, and the resulting mixture was sonicated for 5 min. The concentration of piroxicam in the obtained solution was determined based on the spectrophotometric measurements (λ = 358 nm). The entrapment efficiency (EE) was determined from the Equation (5):

(5) $$EE\left(\%\right)=\frac{weight\;of\;piroxicam\;in\;1\;mg\;of\;the\;pellet}{initial\;weight\;of\;piroxicam\;added\;per\;1\;mg\;of\;the\;pellet}\;\cdot 100$$

Based on Equation (6), the loading efficiency (LE) was also calculated:

(6) $$LE\left(\%\right)=\frac{weight\;of\;piroxicam\;in\;the\;pellet}{weight\;ofthe\;pellet}\;\cdot 100$$

### 3.9. Piroxicam Release Profile from CPNs-PIX

Three series of aqueous solutions of CPNs-PIX particles were prepared (each series consisted of eleven 2 mL samples, each containing 0.05 mg of piroxicam). 2 mL of oleic acid was added to each sample, and all samples were incubated at 37 °C with constant agitation (50 rpm). One sample from each series was taken at the appropriate time intervals, and the absorbance of the oleic phase was measured at λ = 358 nm. The amount of piroxicam released was calculated using the Beer–Lambert law.

### 3.10. Biological Studies

Murine fibroblasts (line NIH3T3, ATCC, CRL-1658) were cultured in MEM (high glucose) medium in a humidified incubator (37 °C; 5% CO_2_). The medium was supplemented with streptomycin (100 U/mL), penicillin (100 μg/mL), and 10% of FBS. Cells were sub-cultured (every 2 days) until the appropriate cell number was obtained. Cells were trypsinized when the plate was 80% confluent, then seeded on the 96-well plates.

50 mg/mL stock solutions of polymers (C-CUR, CUR, A-HPC, and HPC) in PBS were prepared, which were then added to the culture medium to obtain resulting solutions of: 0.1, 0.2, 0.3, 0.4, 0.5, 0.6, 0.7, 0.8 and 1.0 mg/mL. pH of all solutions was the same as in the growth medium. 10 mM solution of piroxicam in DMSO was prepared and then diluted with MEM to obtain the appropriate concentration. MTT assay was used to evaluate the cytotoxicity of piroxicam and polysaccharide derivatives in the NIH3T3 line [47,48]. 8 × 10^4^ fibroblasts were seeded in each well of the 96-well plate and 100 µL of MEM containing the polymer was added. In addition, fibroblasts were stimulated with CPNs and CPNs-PIX systems, as well as with piroxicam in DMSO. After the cells were stimulated, they were incubated for 24 h before MTT test.

24 h after stimulation, the fibroblasts were washed with PBS, and MTT dye was added. The cells were then incubated for 90 min. The formazan crystals formed in each well were dissolved in 5 mM HCl solution in isopropanol and incubated for 30 min at 37 °C. Then absorbance measurements were done at 570 nm using a SpectroStar Nano microplate reader (BMG Labtech, Germany). The results were corrected using a background measurement at 630 nm. The results represent a mean of three independent experiments, each carried out in triplicate. The standard deviation was calculated for each value, and the Student’s t-test was used to compare the groups.

Cells stimulated with CPNs-PIX were also visualized using a fluorescence microscope (Nikon Ti-E inverted microscope equipped with a Nikon A1 confocal system; a 405 nm diode laser beam was used for excitation). The samples for microscopic measurements were prepared by fixing the cells in 1% paraformaldehyde in PBS. Images were captured in emission mode with a 60× magnification. Fluorescent images of piroxicam taken up by the cells were recorded using a 32-channel spectral detector, using a 458 nm barrier filter to remove fluorescent background from the cells.

## 4. Conclusions

The cationic derivative of curdlan (C-CUR) was successfully synthesized and characterized. C-CUR was then mixed with an anionic derivative of hydroxypropylcellulose, A-HPC, to synthesize a series of coacervate nanoparticulate systems which were formed by a spontaneous and immediate self-assembly process in water. Based on DLS measurements, the most promising system, CPNs, obtained at C-CUR: A-HPC weight ratio of 1:25, was selected for further studies. CPNs had an average diameter of 200 nm and a regular, spherical shape. The nanoparticles were shown to be thermoresponsive and exhibited LCST at 41 °C, the temperature relevant for biomedical applications. Piroxicam was then successfully entrapped in the CPNs. The release profile of the drug from CPNs-PIX system was almost linear for the first 2 h and slower afterwards, with 98% of PIX released after 6 h. Biological studies have revealed that while C-CUR shows cytotoxicity at ca. 0.1 mg/mL, most probably to the presence of cationic groups in its chain, neither CPNs nor CPNs-PIX show any cytotoxicity at concentrations as high as 1 mg/mL. Additionally, CPNs-PIX nanoparticles were effectively taken up by cells within 24 h.

The CPNs-PIX system is easily formed in water in a spontaneous, instant process and is characterized by high encapsulation efficiency of piroxicam, beneficial release profile, lack of cytotoxicity, and effective uptake by cells. We therefore propose this new system as a promising carrier to deliver piroxicam efficiently and safely to the patients.

## Figures and Tables

**Table 1:** Mean hydrodynamic diameter (d_Z_), polydispersity index (PDI), and zeta potential (ζ) of various C-CUR/A-HPC systems obtained at different polycation/polyanion ratios.

C-CUR: A-HPC (*w*/*w*)	d_z_ (nm)	PDI	ζ (mV)
1:1	908 ± 54	0.751	13.6 ± 0.5
1:2	856 ± 160	0.859	15.7 ± 0.8
1:3	519 ± 89	0.652	11.2 ± 0.6
1:4	351 ± 30	0.860	14.7 ± 1.5
1:5	909 ± 54	0.751	13.6 ± 0.5
1:10	421 ± 10	0.793	6.2 ± 0.9
1:14	405 ± 84	0.572	−0.1 ± 0.3
1:20	315 ± 2	0.223	−1.0 ± 0.3
**1:25**	**293 ± 1**	**0.197**	**−3.1 ± 0.4**
1:30	260 ± 3	0.260	−4.2 ± 0.4

**Table 2:** LCST (or UCST) values for polysaccharides, their modifications, and obtained nanoparticulate systems in water.

Polymeric System	LCST (L)/UCST (U) (°C)
HPC	43 (L)
A-HPC	43 (L)
CUR	47 (U)
C-CUR	not observed
C-CUR/A-HPC (1:25)	41 (L)

**Table 3:** Mean hydrodynamic diameter (d_Z_) and polydispersity index (PDI) of the C-CUR/A-HPC (1:25) system in various temperatures.

Temperature (°C)	d_z_ (nm)	PDI
21	265 ± 2	0.234
24	256 ± 1	0.218
25	268 ± 2	0.195
27	256 ± 1	0.207
30	254 ± 1	0.218
33	254 ± 1	0.192
36	249 ± 3	0.194
39	239 ± 2	0.192
42	269 ± 1	0.184
45	401 ± 12	0.114
46	861 ± 31	0.259

**Table 4:** Parameters and coefficients received for different models fitted to the experimental piroxicam release profiles from CPNs-PIX systems at various temperatures.

Model	25 °C	37 °C	45 °C
Higuchi			
k_H_	27.3 ± 1.43	22.1 ± 2.76	57.3 ± 2.57
R^2^	0.9306	0.9411	0.9534
Peppas			
a	21.4 ± 1.67	57.3 ± 2.33	53.3 ± 3.02
n	0.594 ± 0.097	0.304 ± 0.031	0.427 ± 0.048
R^2^	0.8991	0.9377	0.9302
Weibull			
a	44.1 ± 13.29	88.7 ± 1.27	97.4 ± 10.87
b	0.99 ± 0.335	0.94 ± 0.061	0.90 ± 0.153
K	0.72 ± 0.443	0.99 ± 0.034	0.96 ± 0.25
R^2^	0.9399	0.9985	0.9819

## Supplementary Materials

Supplementary materials can be found at https://www.mdpi.com/1422-0067/21/24/9664/s1.

## Abbreviations

CUR	Curdlan
C-CUR	Cationic curdlan
HPC	Hydroxypropyl cellulose
A-HPC	Anionic hydroxypropyl cellulose
CPNs	Coacervate polysaccharide nanoparticles
PIROX	Piroxicam
DLS	Dynamic light scattering
AFM	Atomic force microscopy
SEM	Scanning electron microscopy
XPS	X-ray photoelectron spectroscopy
