# Supplementary material for: Coacervate Thermoresponsive Polysaccharide Nanoparticles as Delivery System for Piroxicam
Source: Int J Mol Sci. 2020 Dec 18;21(24):9664. doi: 10.3390/ijms21249664 (PMC7767055; doi:10.3390/ijms21249664)
Supplement: Supplementary file 1 [file ijms-21-09664-s001.pdf]

International Journal of *Molecular Sciences*

MDPI

Supplementary

Coacervate Thermoresponsive Polysaccharide Nanoparticles as Delivery System for Piroxicam

Dorota Lachowicz [1,*], Agnieszka Kaczyńska [2], Anna Bodzon-Kulakowska [3], Anna Karewicz [2], Roma Wirecka [1,4], Michał Szuwarzyński [1] and Szczepan Zapotoczny [2]

[1] Academic Centre for Materials and Nanotechnology, AGH University of Science and Technology, al. A. Mickiewicza 30, 30-059 Krakow, Poland; roma.wirecka@gmail.com (R.W.); szuwarzy@agh.edu.pl (M.S.)
[2] Faculty of Chemistry, Jagiellonian University, Gronostajowa 2, 30-387 Krakow, Poland; agnieszka.kaczynska01@gmail.com (A.K.); karewicz@chemia.uj.edu.pl (A.K.); zapotocz@chemia.uj.edu.pl (S.Z.)
[3] Department of Analytical Chemistry and Biochemistry, Faculty of Materials Science and Ceramics, AGH University of Science and Technology, al. A. Mickiewicza 30, 30-059 Krakow, Poland; anna.bodzon-kulakowska@agh.edu.pl
[4] Faculty of Physics and Applied Computer Science, AGH University of Science and Technology, al. A. Mickiewicza 30, 30-059 Krakow, Poland
* Correspondence: dorota.bielska@agh.edu.pl

Received: 18 November 2020; Accepted: 15 December 2020; Published: date

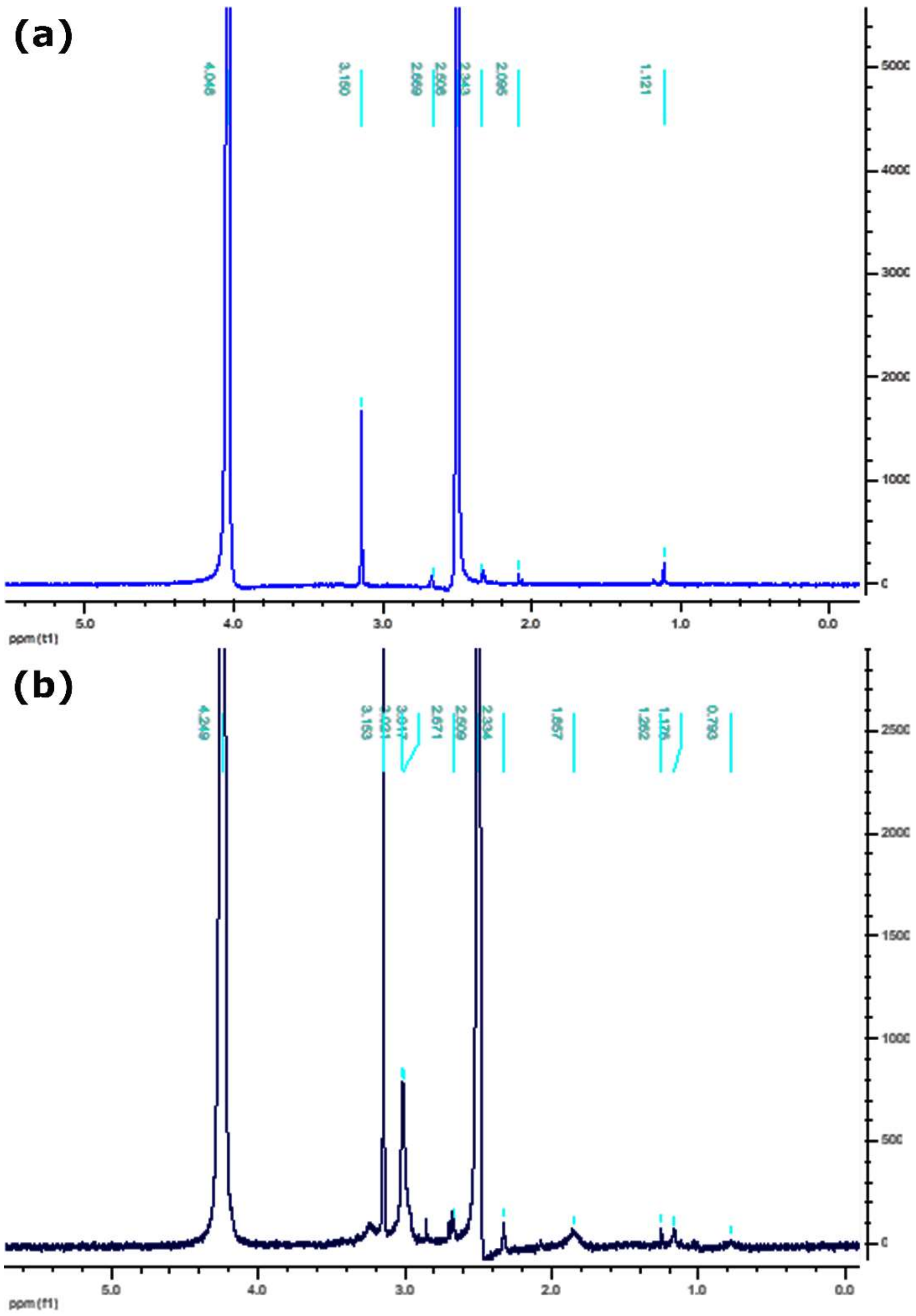

Figure S1. ^{1}H-NMR spectra of CUR (**a**) and C-CUR (**b**) (c = 1.5 mg/mL in D_2O)

Table S1. Substitution degrees for the cationic curdlan (C-CUR).

Material	Degree of substitution (DS %) (based on ^{1}H NMR spectrum)	Degree of substitution (DS %) (based on conductivity measurements - Cho method)
C-CUR	*8.9 ± 1.98*	*11.2 ± 1.11*

Table S2. Substitution degrees for the anionic hydroxypropylcellulose (A-HPC).

Material	Degree of substitution (DS %) (based on elemental analysis)	Degree of substitution (DS %) (based on XPS spectrum)
A-HPC	*29.0 ± 9.81*	*19.2 ± 2.21*

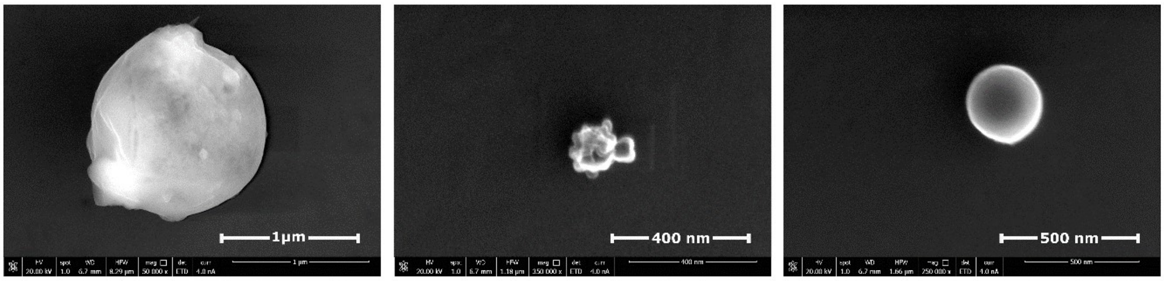

Figure S2 SEM images of the particles obtained for various C-CUR/A-HPC compositions. From the left: C-CUR/A-HPC ratio of: 1:1, 1:10 and 1:25.
